# Exploring the Digital Economy Innovation in the Yangtze River Delta: A Perspective of Complex Networks

**DOI:** 10.3390/e27121241

**Published:** 2025-12-08

**Authors:** Luyun Sun, Pan Zhao, Benda Zhou

**Affiliations:** 1 College of Finance and Mathematics, West Anhui University, Lu’an 237012, China; cloudsta@foxmail.com; 2 College of Economics and Management, West Anhui University, Lu’an 237012, China

**Keywords:** digital economy innovation, spatial correlation, complex network, institutional distance

## Abstract

As a major economic engine of China, the Yangtze River Delta (YRD) region is pivotal in driving innovation across the scientific, technological, and digital economies. This study constructs the spatial associative network for digital economy innovation by treating 41 cities as nodes and applying a gravity model adjusted for institutional distance. Subsequently, the structural characteristics of the spatial associative network and their effects were empirically explored by using complex network analysis and regression models. The findings indicate that: (1) The linkages in digital economy innovation among cities are becoming increasingly closer, and the network structure exhibits an annual increasing trend in density, connectivity, and efficiency, while hierarchy decreases; (2) The examination of network node characteristics discloses that different cities possess diverse capabilities in terms of resource aggregation, regulation, and communication. The block model analysis further categorizes the cities into four functional groups. Among them, Block I (including cities like Shanghai, Nanjing, and Hangzhou) holds the “primary” status and acts as the “core city” for digital economy innovation; (3) The attributes of the spatial associative network have a remarkable effect on both the degree of digital economy innovation and the variations among cities.

## 1. Introduction

Characterized by digitalization, networking, and intelligence, the digital economy is the latest form of modern economic development. It is also the main direction in which the traditional economy undergoes transformation and upgrading. Tapscott initially introduced the concept of the “digital economy” as an economic activity based on digital technologies, especially Internet technologies [1]. In an era of accelerating globalization and digitalization, the digital economy is profoundly transforming global competition. It serves as a key catalyst for industrial transformation and a central driver of economic development [2,3].

As a pivotal hub for China’s modernization, the Yangtze River Delta (YRD) has seen its strategic significance and innovative capacity grow increasingly prominent. China aims to establish seven National Pilot Zones for Digital Economy Innovation, three of which are located in the YRD—Shanghai, Jiangsu, and Zhejiang—underscoring the region’s central role in the national digital development strategy [4]. Since the launch of the Digital Yangtze River Delta Development Plan in 2021, the YRD has maintained a leading position in digital development among China’s four major economic clusters: the YRD, Beijing-Tianjin-Hebei, the Pearl River Delta, and Chengdu-Chongqing [5]. By the end of 2024, the YRD had constructed over 820,000 5G base stations, accounting for nearly one-fifth of the national total. It is also home to 32 “Gigabit Cities” and leads the nation in total intelligent computing power. Driven by the rapid expansion of artificial intelligence (AI) enterprises such as DeepSeek and iFlytek, the region’s influence in AI has steadily grown. In 2024 alone, the region recorded 364,000 newly registered digital economy enterprises—the highest among China’s four major economic clusters and accounting for 23.4% of the national total [6]. Leveraging its economies of scale, technological innovation capabilities, policy synergies, and talent agglomeration, the YRD has emerged as a core growth engine and innovation pacesetter in China’s digital economy, serving as a key benchmark and catalyst for digital transformation in China and even globally.

As information technology develops rapidly, the links between cities have grown increasingly tight, and the flow of digital economy innovation elements within the region has become more frequent, exhibiting significant spatial correlation [7,8]. This spatial correlation is not disorganized but has formed a complex network structure, which contains rich information and patterns. A thorough exploration of the spatial associative network structure and its impacts on digital economy innovation helps in understanding the internal mechanism of regional digital economy innovation. Moreover, it plays an important role in optimizing resource distribution, fostering collaborative innovation, and driving the development of regional integration. Specifically, understanding the network structure helps clarify each city’s position and role in digital economy innovation. Different cities, relying on their unique resource endowments, industrial bases, and innovation capabilities, play different roles in the network. Some cities have become centers for gathering innovation resources, while others occupy key positions in information transmission and resource integration, and still others are in a relatively marginal state. Precisely identifying these roles contributes to cities’ formulation of accurate digital economy development strategies, allowing each city to fully utilize its own advantages and achieve differentiated development. In addition, examining the influences of the network structure on digital economy innovation, whether on the improvement of innovation levels or the impact on urban discrepancies, is beneficial to the formulation of regional policies, thus achieving a more just and efficient digital economy development pattern.

Hence, this study chooses 41 cities in the YRD as network nodes. It utilizes the gravity model modified by institutional distance to construct a spatial associative network of digital-economy innovation. By means of complex network analysis techniques, it comprehensively investigates its structural features. Regression analysis reveals the influence of the network structure on digital economy innovation.

## 2. Literature Review

### 2.1. The Digital Economy Innovation and Spatial Spillovers

Digital economy innovation refers to economic activities that utilize core digital technologies—such as big data, AI, cloud computing, and the Internet of Things—to develop new products, processes, and business models, thereby improving total factor productivity [9]. Spatial spillovers significantly influence regional innovation dynamics and economic development [10]. A growing body of regional innovation literature has turned its attention to cross-regional spillover effects. Wang (2025), employing spatial econometric methods such as the Spatial Durbin Model (SDM), demonstrated that digital economy innovation generates positive spatial spillovers through channels including technology diffusion and industrial upgrading, thereby significantly driving growth in neighboring regions [11]. In contrast, Barabuffi et al. (2025) identified a negative interprovincial spillover effect of artificial intelligence (AI) patents in China, which exacerbates technological and innovation disparities across regions and consequently impairs national-level AI patent performance [12].

From a methodological perspective, researchers have widely adopted spatial econometric tools—constructing spatial weight matrices primarily based on geographical adjacency or inverse geographical distance—to empirically examine the impact of the digital economy or digital innovation on economic growth, green total factor productivity, and carbon emission performance [13,14,15]. This has given rise to an “attribute-based” analytical paradigm. The spatial econometric framework offers a more effective approach for addressing causal inference in spatial data [16]. It enables the quantification of the magnitude and direction of spillover effects, disentangles the net impacts of different attribute variables through regression coefficients, and provides robust evidence regarding the loci of spillovers [17].

However, this methodology, while confirming the existence and strength of spatial associations, often fails to reveal their concrete structure and underlying mechanisms [18]. Critical questions regarding the specific pathways of innovation diffusion or the distinct roles of regions within the network remain unaddressed, creating a “black box” in spatial econometric models. In comparison, complex network analysis offers a novel “relationship-based” perspective. This paradigm treats cities or regions as “nodes” and the economic or innovation linkages between them as “edges”, analyzing relational data directly to uncover the overall structure and internal patterns of spatial systems [19].

Currently, a growing number of researchers are adopting this complex network approach to analyze spatial interconnections in the digital economy across regions, with a particular focus on the YRD in China. For instance, Chu et al. (2023) suggest that the spatial network of the digital economy among cities in the YRD has basically formed, albeit with a relatively low network density [20]. Xu et al. (2022) believe that the spatial correlation of digital economy development among cities in the YRD is becoming closer, but the correlation network is unstable [21]. Additionally, Fan et al. (2023) found that by examining the spatial correlation of industrial chain agglomeration, the development of the digital economy can significantly accelerate the industrial chain upgrading pace in the YRD [22]. However, as Lai et al. pointed out, although the development level of the digital economy will remain stable in the next two years, the gradual slowdown of its development will bring it close to a relatively stagnant period [23]. In this context, exploring digital economy innovation becomes especially crucial. As the core driving force of digital economy development, digital economy innovation plays an irreplaceable and critical role in promoting regional economic growth and enhancing overall competitiveness.

### 2.2. Construction of the Spatial Connectedness Network

Identifying the spatial connectedness relationships serves as the foundation for complex network analysis. According to the extant literature, the principal methods for determining spatial correlation relationships encompass the modified gravity model, Vector Autoregression (VAR) Granger test, time-lag correlation coefficient, etc. [24,25,26]. Among the three methods, the VAR model is overly sensitive to the selection of its lag order, which somewhat undermines the reliability of the network structural features derived from it. Moreover, the VAR-based network construction fails to adequately capture the evolutionary trajectory of the spatial connectedness network. Furthermore, both the VAR model and the time-lag correlation coefficient approach require relatively large sample sizes. In contrast, the modified gravity model effectively addresses these limitations.

In terms of research methods, the gravity model has been extensively utilized to compute the economic connection intensity within urban agglomerations. This is particularly evident in regions such as the Beijing-Tianjin-Hebei, YRD, and Pearl River Delta [27,28,29]. For the purpose of better reflecting the complex connections between cities, some revised gravity models have gradually emerged. For example, some research has introduced the time-distance variable to replace the traditional physical distance to more accurately consider traffic convenience and cost [30]. In fact, due to the “time-space compression” caused by the development of information technology and transportation technology [31], the conventional geographical distance can no longer adequately describe the actual impediments and impacts encountered by the flow of digital economy innovation elements among regions. Factors such as administrative barriers and institutional differences, which constitute a more complex institutional distance [32], have gained increased importance. This institutional distance is intertwined with the geographical distance, jointly affecting the spatial correlation of digital economy innovation between cities. It is noteworthy that existing research has demonstrated that the adoption of digital technology significantly facilitates inter-city innovation collaboration, transcending geographical distance and urban hierarchy boundaries. This further underscores the necessity of incorporating multiple forms of distance in constructing spatial connectedness networks [33]. For instance, discrepancies in policies, regulations, and standards regarding the digital economy among different regions may cause varying degrees of attenuation or distortion when production factors from one region spread to another. This, in turn, changes the flow direction and efficiency of digital economy innovation resources. Consequently, depending only on the traditional geographical distance for constructing a spatial associative network model fails to precisely represent the spatial connection status in reality, and there is an immediate necessity to include distance factors from more dimensions. This is to enhance the scientific nature and effectiveness of the construction and analysis of the spatial associative network of digital economy innovation.

In summary, this study makes several contributions compared to the existing literature. First, from a methodological perspective, it shifts the research on digital economy innovation from an “attribute-based” to a “relationship-based” paradigm and attempts to integrate both approaches. Employing complex network analysis addresses the limitations of econometric methods in revealing intricate structural patterns. Furthermore, it utilizes panel regression models to examine how both network structural features and node characteristics influence key attributes. Second, regarding analytical scale, while most current studies on digital economy innovation focus on national or provincial levels, relatively few delve into micro-level analyses such as urban agglomerations. This research enriches micro-scale analysis by concentrating on the YRD urban cluster in China. Simultaneously, by focusing on China’s national strategy of integrated development in the YRD, the findings offer targeted decision-making support and practical guidance for breaking down administrative barriers and optimizing the layout of regional digital innovation. More importantly, in model construction, this study introduces “institutional distance” as a core variable into a gravity model to build the spatial connectedness network. This approach ensures that the constructed “relational” data better aligns with the inherent nature of digital economy innovation, which often transcends administrative boundaries, thereby enhancing the theoretical validity of the network model.

## 3. Materials and Methods

### 3.1. Research Region and Data

The YRD, which is situated on the eastern coast of mainland China, is one of the regions with a high degree of regional cohesion in China. The YRD region includes three provinces and one directly administered municipality, namely Jiangsu Province, Zhejiang Province, Anhui Province, and Shanghai. This region comprises a total of 41 cities and has an area of 358,000 km^2^. This is slightly larger than the area of Germany (357,000 km^2^) and marginally smaller than that of Japan (378,000 km^2^). The YRD is divided into six metropolitan areas (See Table 1). In 2023, the total economic output of the YRD reached 30.5 trillion yuan. Among this, the added value of the digital economy surpassed 12 trillion yuan, accounting for about 40% of the YRD’s GDP and 30% of the added value of the national digital economy [34].

In this research, 41 cities within the YRD are considered as the analytical objects. The sample period spans from 2010 to 2020. The main data utilized in this research is the digital economy innovation index (DEI) data, which is derived from the Index of Regional Innovation and Entrepreneurship in Digital Economy in China (IRIEDEC) dataset [35]. IRIEDEC is a system designed to scientifically evaluate the development level of the digital economy across various regions in China. It focuses on the innovation and entrepreneurship behaviors of enterprise entities, with key coverage of activities in the four core industries of the digital economy. Relying on the enterprise big database integrated and constructed by the Enterprise Big Data Research Center of Peking University, the index comprehensively assesses the regional development level of the digital economy from three dimensions: enterprise entry, investment activities, and innovation outputs. Specifically, through six dimensions (including the number of new enterprises, attractiveness of foreign investment, attractiveness of venture capital, number of authorized patents, number of registered trademarks, and number of registered software copyrights) consisting of a total of eight indicators, the index measures the intensity of innovation and entrepreneurship activities in the digital economy field of China’s provincial and municipal administrative regions from 2010 to 2020, providing an accurate measurement of the innovation and entrepreneurship performance of the digital economy in various regions. The index has a value range of 0–100, with higher values indicating a higher level of digital economy innovation and entrepreneurship development in the region.

### 3.2. The Adjusted Gravity Model

The spatial associative network is a collection of relationships among cities. Each city acts as a “node” of the network, and the spatial correlation relationships among cities are the “edges”. These nodes and edges together constitute the spatial network structure. The crux of constructing the spatial associative network is to depict the spatial correlation relationships among cities, for which the adjusted gravity model is employed. The gravity model has become a frequently used model for studying urban spatial linkages [36], and its basic form is:

(1) $$R_{ij}=k_{ij}\frac{M_{i}M_{j}}{D_{ij}^{r}}$$

In the formula, *R_ij_* stands for the strength of digital economy innovation connection from city *i* to city *j*, which is termed as the digital economy innovation connection quantity; *M_i_* and *M_j_* denote the digital economy innovation index (DEI) values of cities *i* and *j*, respectively. The generalized index method is adopted to standardize the percentage system to the value range of 0 to 1; *D_ij_* represents the distance between city *i* and city *j*; *k_ij_* is the gravitational coefficient; *r* is named the friction coefficient, and its value is usually 1. Taking into account the asymmetry of the digital economy innovation connection among cities, the formula for *k_ij_* is as follows:

(2) $$k_{ij}=\frac{M_{i}}{M_{i}+M_{j}}$$

Institutional distance exerts a significant influence on the flow of digital innovation factors, making it essential to incorporate this dimension into the gravity model. Institutions, defined as humanly devised constraints that structure social interaction, constitute the rules of the game in a society [37]. Building on Scott’s “three pillars” framework [38], Kostova (1996) first introduced the concept of institutional distance, defining it as the extent of dissimilarity between the institutional environments—economic, political, and cultural—of different countries or regions [39]. Institutional distance can generally be categorized into formal and informal institutional distance, or further distinguished as regulatory, normative, and cognitive distance [40]. It affects the cross-regional flow of digital innovation factors—such as knowledge, technology, talent, and data—through the following mechanisms:

First, transaction costs and uncertainty. Greater institutional distance increases transaction costs related to contract enforcement, intellectual property protection, and regulatory compliance in cross-regional collaboration [41]. Concurrently, institutional misalignment elevates market and policy uncertainty, discouraging long-term corporate investment in innovation factors [37,42]. Second, knowledge transfer and absorptive capacity. Significant institutional distance impedes the transfer of tacit knowledge, owing to differences in cognitive frameworks, technical standards, and industry practices [43,44]. Even when technical elements flow into a region, they may not be effectively absorbed if the local institutional environment—such as supporting regulations or talent systems—is underdeveloped [45,46]. Third, network and platform effects. Digital innovation thrives on open platforms and collaborative networks. Regions with institutional proximity are more likely to establish shared data norms, mutual trust mechanisms, and compatible technical interfaces, thereby facilitating the movement of innovation factors [47]. Fourth, institutional escape. Excessively large institutional gaps may also lead to “institutional escape”—where firms withdraw to their home markets due to difficulties in adapting to host institutional conditions—thereby hindering two-way flows of resources [48,49].

Therefore, it is more appropriate to adopt a comprehensive distance measurement method that introduces institutional distance. This approach not only considers the traditional geographical distance but also includes the institutional distance, allowing for a more comprehensive evaluation and comprehension of the economic interaction and integration degree between different cities. The revised distance formula is as follows:

(3) $$D=w_{1}D_{1}+w_{2}D_{2}.$$

Among them, *D*_1_ is the geographical distance, which is denoted by the spherical distance calculated from the longitudes and latitudes of the two cities’ municipal governments; *D*_2_ is the institutional distance, which is measured by the boundary institutional distance. Considering the administrative division boundary (rigid regional division) and the metropolitan area boundary (non-rigid regional division), the institutional distance variable is defined as follows: 1 = within the same province and the same metropolitan area; 2 = in the same province but not belonging to the same metropolitan area; 3 = in different provinces but belonging to the same metropolitan area; 4 = in different provinces and not belonging to the same metropolitan area. Due to the dynamic change of the planning scope of the metropolitan area, the institutional distance between the two cities may vary from year to year. Assuming that the geographical distance and the institutional distance have an equal degree of restriction on the connection between cities, both *w*_1_ and *w*_2_ are set to 0.5.

Both *D*_1_ and *D*_2_ are standardized using the generalized index method, with the formula presented below:

(4) $$D^{’}=\frac{D}{maxD}$$

Based on Formulas (1)–(4), this paper computes the gravity matrix of digital economy innovation to reflect the correlation among cities in the YRD. To make the outcomes comparable across different time periods, this paper takes the mean value of the elements of all gravity matrices from 2010 to 2020 as the threshold. If the gravitational value exceeds this threshold, it is recorded as 1, indicating that there is a correlation between the city in this row and the city in this column in terms of digital economy innovation. On the contrary, if the gravitational value is below the threshold, it is recorded as 0, indicating that there is no such correlation. Thus, a binarized gravity matrix is obtained for further analysis.

In constructing the spatial connectedness network of inter-city digital economy innovation, this study adopts a bidirectional directed network framework, which differs from the undirected or unidirectional directed networks commonly used in the literature. As shown in Equations (1) and (4), the connection strength between any two cities, *i* and *j*, is calculated independently for each direction. Therefore, when the connection strength in both directions exceeds the predefined threshold, the network will contain directed edges in both *i*→*j* and *j*→*i* directions. This design stems from our understanding of the innovation spillover process: the flow of innovation factors such as knowledge, technology, and talent is not a unidirectional “center-periphery” diffusion, but involves complex bidirectional interactions. For instance, while Shanghai may transfer technology to Hefei, Hefei may in turn contribute knowledge back to Shanghai, leveraging its strengths in scientific talent. Thus, the use of a bidirectional directed network enables a more authentic and granular representation of the asymmetric yet reciprocal linkages between cities, thereby providing a solid structural basis for analyzing the diversity of nodal roles.

### 3.3. Complex Network Analysis

#### 3.3.1. Network Structural Features

Network density, which varies from 0 to 1, is the proportion of the number of actual correlation links in the network to the maximum possible number of correlation links in the whole network. This indicator reflects the density of the correlative links among cities in the network. The greater the network density, the more correlative links there are in the network, and the closer the connections among cities. The computational formula is as follows [50]:

(5) $$ND=\frac{r}{n(n-1)}.$$

*ND* represents the network density, *r* is the number of actual correlation links, and n is the number of cities.

Network connectedness reflects the robustness and vulnerability of the network [51]. It can be denoted by the extent to which any two nodes in the network are directly or indirectly accessible, varying from 0 to 1. The larger this indicator is, the stronger the accessibility of the network and the more robust the network relationships will be [52]. The computational formula is as follows:

(6) $$NC=1-\frac{m}{n(n-1)}.$$

*NC* stands for the network connectivity, *m* is the number of inaccessible city pairs in the network, and n is the number of cities.

Network hierarchy reflects the degree to which cities in the network are asymmetrically accessible, varying from 0 to 1. The larger this indicator is, the more hierarchical the structure among cities in the network is, and the more cities are in a subordinate and marginal position in the spatial associative network. The computational formula is as follows [52]:

(7) $$NH=1-\frac{K}{max(K)}.$$

*NH* stands for the network hierarchy, *K* is the number of symmetrically accessible city pairs, and max(*K*) is the maximum possible number of symmetrically accessible city pairs in the network.

Network efficiency reflects the circulation of elements within the network and the responsiveness to external disturbances. It is measured by the global efficiency [53] and the computational formula is as follows [54]:

(8) $$NE=\frac{1}{n(n-1)}\sum_{i\neq j}\frac{1}{d_{ij}}.$$

*NE* represents the network efficiency; n is the number of cities; and *d_ij_* represents the shortest route between city *i* and city *j*. Network efficiency quantitatively reflects the average efficiency of information exchange among cities in the network, ranging from 0 to 1. The greater this indicator is, the more linkages there are among cities in the network, the more intricate the correlations among cities are, the more stable the spatial network is, and the higher the efficiency of information exchange in the network will be. This also implies that the spatial associative network can more readily facilitate the spatial spillover of urban digital economy innovation and reduce the disparity of comparative advantages among cities.

#### 3.3.2. Network Node Features

Degree centrality describes the degree to which each city radiates outward and receives external radiation. In a directed graph, edges have directions, which leads to the need to consider the connection situation of nodes from two directions. Therefore, it can be classified into in-degree centrality and out-degree centrality. Among them, out-degree centrality denotes the number of edges originating from a node. The larger the value, the greater the influence and radiation scope of the node. In-degree centrality signifies the number of edges directed at a node from other nodes. The larger the value, the higher the extent to which the node receives external radiation. The computational formula for degree centrality is as follows [55]:

(9) $$C_{d}=\frac{l}{n-1}$$

*C_d_* represents degree centrality, l is the number of cities directly associated with a certain city, and n is the number of cities.

Betweenness centrality gauges the degree to which a city is “in the middle” of other cities, namely, it depicts the extent of control of a city over the correlations among other cities. This indicator ranges from 0 to 1. The larger this indicator is, the stronger the city’s control effect on other cities and the more obvious its core position will be. The computational formula is as follows [56]:

(10) $$C_{b}=\sum_{i\neq j\neq k}\frac{g_{jk}(i)}{g_{jk}}$$

*C_b_* represents betweenness centrality, where *j*, *k*, and *i* represent different cities. *g_jk_* is the number of geodesic paths from city *j* and city *k*, and *g_jk_*(*i*) is the number of shortcuts between city *j* and city *k* passing through city *i*.

Closeness centrality depicts the level of direct connection of a city with other cities, that is, the extent to which the city is “not dominated by other cities” in the network. The greater the value of this indicator, the more direct connections there are between the city and other cities, the shorter the shortcut distances, and the more likely the city is to be a central entity in the network. The computational formula is as follows [57]:

(11) $$C_{c}=\frac{n-1}{\sum_{j=1}^{n}d_{ij}}$$

*C_c_* represents closeness centrality, *d_ij_* is the shortcut distance between city *i* and city *j*, and *n* is the number of cities. In a directed graph, the closeness centrality can be taken into account from the perspectives of “in” and “out”, respectively. Thus, closeness centrality can be categorized into out-closeness centrality and in-closeness centrality.

#### 3.3.3. Network Group Features

Block model analysis was initially proposed by White et al. [58]. It offers a significant methodological foundation for social network analysis and uncovers the roles and positional relationships within the network structure by categorizing and describing the nodes in the network. A block model assigns the nodes in a network to various positions, and each node in each position has structural equivalence. It can be said that the block model is a study at the position level. Burt conducted a classification study on positions. His classification method considers both whether the relational connections mainly emerge within a position and whether the connections are pointed towards the position from other places [59]. On the basis of Burt’s research, Wasserman & Faust (1994) improved the classification of positions [57].

In accordance with the relationships within and between positions, four types of position typologies can be identified, namely, primary, broker, isolate, and sycophant position (See Table 2). As shown in Table 2, *g* represents the total number of economic entities; *B_k_* is a specific position identifier within the network. *g_k_* indicates the number of economic entities at position *B_k_*. *x_ijr_* is the binary variable indicating the presence or absence of a relational tie from entity *i* to entity *j* on relation type *r*. The columns are related to the first differentiation (whether to receive “choices” or not), and the rows are associated with the second differentiation (the proportion of “choices” within the position).

By utilizing block model analysis, it is possible to investigate the group characteristics of the digital economy innovation spatial associative network from a new perspective and reveal the relationships and connection patterns among blocks. However, as the spatial association of digital economy innovation becomes increasingly close, most urban nodes have become the primary position. The above four-type typology sometimes fails to effectively distinguish among various cities. Therefore, this study puts forward a relative classification approach aiming to analyze the relative roles and features of cities in the spatial association of digital economy innovation. Specifically, a coordinate system is constructed with the receiving “choices” ratio as the abscissa and proportion of “choices” within the position as the ordinate, thus forming four quadrants. According to the relative positions of cities in this coordinate system, quadrant I corresponds to the principal position, quadrant II to the isolated position, quadrant III to the obsequious position, and quadrant IV to the intermediary position, to classify and analyze the characteristics of cities.

Cities in quadrant I (the primary position) can not only efficiently receive external digital economy innovation resources but also possess a robust internal innovation cooperation system. Cities in quadrant II (the isolate position) have a relatively high internal tightness but a low external receiving ratio, and have relatively fewer exchanges with the outside world. Cities in quadrant III (the sycophant position) usually have a relatively low internal tightness and a low external receiving ratio, and their digital economy innovation resources are more likely to be transferred to cities in other blocks, just like they are trying to curry favor with external advantaged cities by transferring their innovation resources to them. Cities in quadrant IV (the broker position) have a relatively high external receiving ratio but a low internal tightness. These cities are adept at serving as bridges and links among the digital economy innovation entities of various cities, facilitating the optimal allocation of resources and the circulation and sharing of information.

### 3.4. Effect Analysis

Structure dictates the performance. Based on describing the network structure features of the spatial correlation of DEI, this paper further examines the influence the network structure of the spatial correlation of DEI has on the “attribute data”. The network structure is examined from two levels, namely the overall level and the individual level, and the impact effects are reflected in two aspects: the level of DEI and urban differences. Firstly, a simple OLS regression model is adopted to analyze the effects of the network structure. Specifically, the mean and standard deviation of each city’s DEI are used as dependent variables, while network density, connectedness, hierarchy, and efficiency are independent variables. Secondly, a panel regression is adopted to analyze the effects of the individual network structure. The digital economy innovation indices for each city during the sample period are taken as the dependent variables, and the degree centrality, closeness centrality, and betweenness centrality for each city are taken as the independent variables.

## 4. Results

### 4.1. Spatial Correlation Characteristics of DEI

#### 4.1.1. Structural Features of the DEI Spatial Network

**Figure 1:**
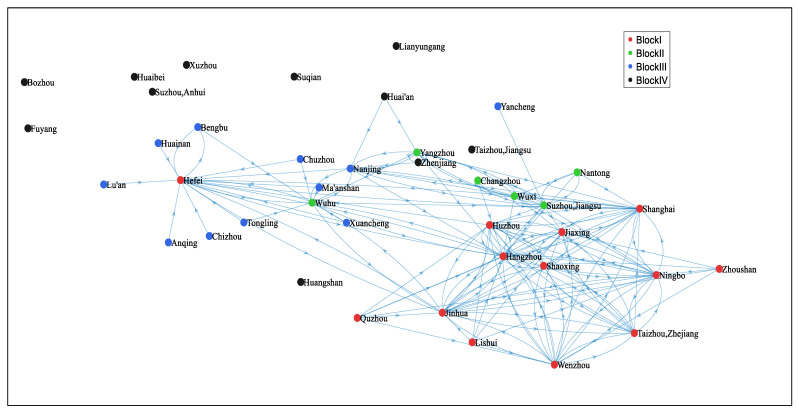
Spatial associative network of digital economy innovation in the Yangtze River Delta, 2010.

**Figure 2:**
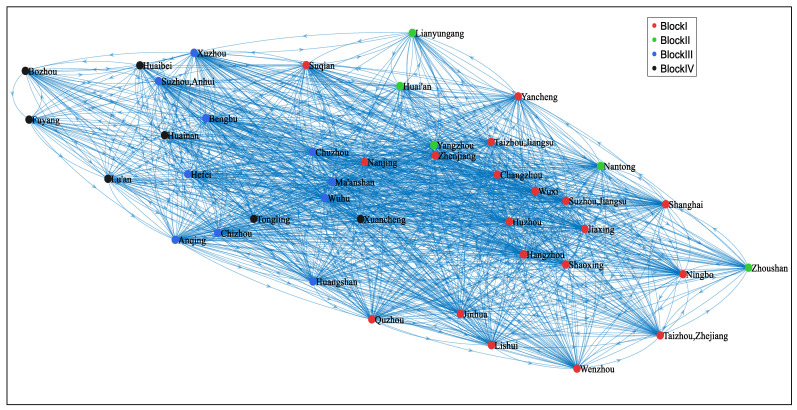
Evolved spatial associative network of digital economy innovation in the Yangtze River Delta, 2020.

Depending on the adjusted gravity model, this study constructs a directed and asymmetric spatial connectedness network of digital economy innovation among the cities in the YRD. To visualize the structure of this network, we employed MATLAB R2020a to draw the out-degree network diagrams for 2010 and 2020, as shown in Figure 1 and Figure 2, which highlight the cities’ outward radiation capacity. It can be observed that the spatial correlation exhibits a typical network structure, with widespread spillover phenomena by 2020.

**Figure 3:**
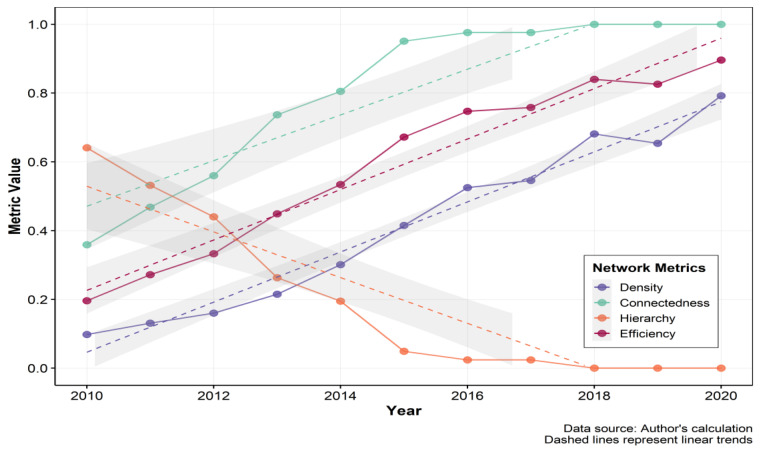
Trends in overall network structure characteristics in the Yangtze River Delta (Multi-indicator line graph, 2010–2020).

As depicted in Figure 3, from 2010 to 2020, the network density of the spatial correlation of DEI displayed an increasing trend annually, rising from 0.098 in 2010 to 0.792 in 2020. The annual increase in network density indicates that the spatial correlation of DEI among cities in the YRD has grown progressively stronger.

Network connectedness, hierarchy, and efficiency can jointly measure the robustness and vulnerability of the network. The results demonstrate that the network connectedness rose from 0.359 in 2010 to 1 in 2018, and remained at 1 after 2018, indicating that the connection of DEI among cities has become closer annually, with a significant improvement in network connectedness and maintaining a high level, demonstrating good accessibility and spatial spillover effects. In addition, the network hierarchy has decreased annually, dropping from 0.641 in 2010 to 0 in 2018 and remaining unchanged after 2018. This result indicates that the previously relatively hierarchical state of the spatial correlation of DEI has been gradually broken, the subordinate relationship among cities has gradually weakened, the status of each city in DEI has become more equal, and the mutual connection and influence have gradually increased. Then the network efficiency has demonstrated an increasing trend annually, rising from 0.196 in 2010 to 0.896 in 2020, which illustrates that the efficiency of information or resource transmission in the network has been continuously improved, and the information transmission capacity of the network has been continuously enhanced. These changes jointly indicate that the connection of DEI has become increasingly close, and the spatial associative network has shown a trend of enhanced robustness and reduced vulnerability. The integration procedure has efficiently facilitated innovative collaboration and resource sharing within the region, providing impetus for the sustainable development of the digital economy in the YRD.

#### 4.1.2. Node Features of the DEI Spatial Network

This part performs network centrality analysis by calculating indicators like degree, betweenness, and closeness centrality, to explore and disclose each city’s status and role in the DEI spatial associative network. For 2020, the calculation results are presented in Table 3.

(1) Degree centrality. From the perspective of in-degree centrality, the mean of centrality in the YRD is 0.792, and there are 29 cities with an in-degree centrality higher than this mean. Among them, the cities with an in-degree centrality of 1 are Nanjing, Hangzhou, Jinhua, Hefei, Wuhu, and Chuzhou, indicating that these cities are the centers for gathering digital economy innovation resources in the YRD, can better attract the innovation elements of other cities in the YRD to flow towards them, and have an absolute advantage in the agglomeration of innovation resources. The cities with a relatively low in-degree centrality are Tongling, Huai’an, Fuyang, Bozhou, Huaibei, and Lianyungang, which indicates that these cities lack the key factors to attract external innovation resources, so few other cities will transfer innovation resources (such as the transfer of scientific research achievements, the inflow of innovative talents, etc.) to these cities. From the perspective of out-degree centrality, there are 26 cities among the 41 cities in the YRD with an out-degree centrality higher than the mean. Among them, Wuhu has the highest centrality, indicating that Wuhu assumes a core role in the output of innovation achievements and the dissemination of technology in the YRD. Fuyang, Bozhou, and Huaibei have relatively low out—degree centralities, and these cities have restricted driving impacts on the digital economy innovation development of other cities in the YRD.

(2) Betweenness centrality. The mean of the betweenness centrality of the 41 cities in the YRD is 8.317. The TOP 5 cities are Wuhu, Chuzhou, Hefei, Ma’anshan, and Nanjing. These cities are important nodes for the circulation of digital economy innovation information and resources in the YRD, playing an indispensable intermediary role in transmitting innovation elements and having a strong control over the resource flow path. The cities with a relatively low betweenness centrality are Lianyungang, Zhoushan, and Huaibei. The betweenness centrality of these cities is zero. This implies that these cities occupy a relatively marginal or isolated position in the transmission path of digital economy innovation resources and information. They have no control over the flow of digital economy innovation resources among cities, and scarcely have any say in the integration of regional digital economy innovation resources.

(3) Closeness centrality. The mean of in-closeness centrality of the 41 cities in the YRD is 0.021. The cities ranking high are Wuhu, Chuzhou, Hefei, Nanjing, Hangzhou, and Jinhua, indicating that these cities can obtain the information and resources of other cities through shorter paths or fewer links and are in the central position of the “supply circle” of information resources. The cities featuring the lowest in-closeness centrality are Huaibei and Lianyungang, each having a value of 0.015. They are in a comparatively weak position when it comes to rapidly acquiring innovation information and integrating innovation resources.

In terms of out-closeness centrality, the cities ranking high are Wuhu, Chuzhou, Ma’anshan, and Xuancheng, indicating that these cities will be more timely and convenient than cities with low closeness centrality when transmitting information such as the latest digital technology research and development achievements and changes in digital economy industry policies. Simultaneously, regarding the resources necessary for digital economy innovation, these cities can actively transfer them to other cities. The cities with the lowest out-closeness centrality are Zhoushan, Fuyang, Bozhou, and Huaibei, suggesting that these cities are relatively less advanced in terms of active information dissemination capabilities and the dominance of resource output.

The digital economy innovation network in the YRD does not conform to a simple “core–periphery” structure with Shanghai as the sole center; rather, it constitutes a multi-center, multi-level complex ecosystem. Nanjing, Hefei, and Hangzhou function as “provincial engines” that integrate their respective provinces; Wuhu and Chuzhou serve as “regional bridges” linking external networks; Shanghai and Ningbo act as “knowledge hubs” attracting key resources; while numerous active secondary nodes collectively form a tightly interconnected innovation network.

First, Nanjing, Hefei, and Hangzhou together form the “provincial engines” that drive innovation within Jiangsu, Anhui, and Zhejiang provinces. All three exhibit a high degree of centrality, indicating their central roles in their respective provincial innovation networks. Among them, Nanjing and Hefei demonstrate stronger “control capacity,” with significantly higher betweenness centrality scores (20.750 and 22.613, respectively) than Hangzhou, suggesting they serve not only as resource hubs but also as “coordination centers” for the flow of innovation resources within their provinces. Hangzhou displays a “high absorption–strong endogenous” characteristic. Its highest in-degree centrality (1.000) reflects a strong capacity to attract resources, while its relatively lower out-degree (0.825) and betweenness centrality (9.906) indicate that its spillover model focuses more on deeply internalizing and transforming absorbed resources within its own provincial and internal systems, rather than acting as a cross-provincial bridge.

Second, Wuhu and Chuzhou stand out as the most distinctive cities, highlighting the “bridging” function that spans structural holes. Both cities rank among the top in out-degree centrality (Wuhu 0.975, Chuzhou 0.950), even surpassing Nanjing and Hefei. This indicates their strong initiative and capability to proactively establish external innovation linkages, making them highly active “radiation sources” in the network. Their betweenness centrality scores (Wuhu 26.314, Chuzhou 24.649) rank first and second in the entire region, providing the strongest evidence of their “bridge” role. This implies that not only within their own province but also in connecting sub-networks of other provinces in the YRD, a significant volume of innovation flows (e.g., technical collaboration, knowledge spillovers) must pass through them. They substantially enhance the integration depth between Anhui and the Jiangsu-Zhejiang-Shanghai region, serving as critical pivots for regional integration in the digital economy.

Third, both Shanghai and Ningbo exhibit high in-degree but relatively low out-degree centrality. Shanghai’s in-degree (0.925) is considerably higher than its out-degree (0.700); Ningbo shows an even more pronounced disparity (in-degree 0.900, out-degree 0.625). This suggests that many cities actively establish links with them, channeling innovation elements toward them, while their initiative in radiating influence outward is relatively limited. Their betweenness centrality scores (Shanghai 2.842, Ningbo 1.531) remain at low levels, indicating that they are not primary hubs for facilitating connections between other cities. Resources flow toward them but seldom need to pass through them to reach other nodes. This phenomenon may reveal the “siphon effect” or “knowledge hub” characteristic of Shanghai as the leading city, and a similar pattern for Ningbo, which likely serves as a key manufacturing and innovation application center, extensively absorbing external resources.

Fourth, a group of cities represented by Suzhou, Wuxi, and Ma’anshan function as active secondary nodes in the network. They generally exhibit a high degree of centrality (both out-degree and in-degree greater than 0.85), indicating they are “active participants” in the network, maintaining close bidirectional interactions with most partners. At the same time, they also possess moderately high betweenness centrality (e.g., Ma’anshan 22.272, Wuxi 9.009), suggesting they play certain intermediary roles within the region or along specific pathways. These cities serve as essential supports for the core hubs and network bridges, forming a solid foundation for the digital economy innovation network of the YRD.

To investigate the economic foundations of cities’ centrality within the network, this study systematically examines the statistical associations between per capita GDP and various network centrality metrics. The per capita GDP data for the 41 cities from 2010 to 2020 were obtained from the official statistical yearbooks of Shanghai, Zhejiang, Jiangsu, and Anhui. Given the panel structure of the data, we estimate their correlations from three distinct dimensions:

(1) Pooled correlation, which ignores individual and time dimensions by treating all observations as independent samples, reflecting the overall co-variation trend between variables;

(2) Between-entity correlation, obtained by calculating the mean value of each indicator for each city over the sample period, capturing the structural relationship between long-term economic levels and network positions across cities;

(3) Within-entity correlation, which examines how a city’s economic performance and network centrality co-vary over time, after controlling for individual fixed effects.

To further ensure the robustness of the results, each type of correlation is estimated using both Pearson’s product-moment correlation and Spearman’s rank correlation. The former assumes a linear relationship, while the latter makes no distributional assumptions and is more capable of capturing monotonic trends.

Table 4 presents the analysis results across the three dimensions: pooled, between-entity, and within-entity correlations. Overall, the direction of Pearson and Spearman correlation coefficients is highly consistent, supporting the robustness of the findings. For ease of interpretation, the following discussion will focus primarily on the Pearson correlation coefficients. The results reveal a multi-layered and differentiated pattern in the relationship between economic strength and network position.

First, economically developed cities generally occupy more central positions in the network. Most centrality metrics show significant positive correlations with per capita GDP across the pooled and between-entity analyses. It is particularly noteworthy that in-degree centrality (pooled correlation = 0.604) and in-closeness centrality (pooled correlation = 0.587) exhibit the strongest associations with economic level, indicating that economically advanced cities have a clear advantage in attracting external innovation factors. This finding corroborates the Matthew effect in economic development—cities with stronger economic capacity attract more knowledge, technology, and talent, thereby consolidating their core position in the network.

Second, long-term economic disparities between cities explain network position differences more effectively than short-term fluctuations. Between-entity correlations are generally higher (e.g., between-entity correlation of out-degree centrality = 0.526), reflecting how persistent economic disparities shape cities’ network positions. In contrast, while within-entity correlations are also significant, their economic implication differs: they indicate that when a city’s economic performance exceeds its own average level, its network centrality also tends to increase. This provides preliminary evidence for an inherent linkage mechanism between economic performance and network position.

Third, betweenness centrality exhibits a unique pattern, revealing the complexity of network structure. The relationship between betweenness centrality and per capita GDP is distinctive: although pooled correlation (0.188, *p* < 0.01) and between-entity correlation (0.381, *p* < 0.05) are positive, they are relatively weak, while the within-entity correlation is significantly negative (–0.231, *p* < 0.01). This seemingly contradictory result carries important economic implications: it suggests that cities experiencing faster economic growth over specific periods may lose their role as “bridges” in the network. A possible explanation is that rapid economic growth may lead cities to focus more on internal resource integration rather than acting as connectors between different groups, or it may reflect complex interactions between network structure evolution and economic cycles.

#### 4.1.3. Network Group Features

The spatial clustering characteristics of each city in the YRD within the digital economy innovation correlation network in 2020 are revealed by using block model analysis. The Concor method is adopted, with the maximum segmentation depth set to 2 and the concentration standard set to 0.2. As a result, the 41 cities in the YRD are divided into four blocks. The division results are presented in Table 5.

Among these blocks, Block I consists of 19 cities, namely Shanghai, Nanjing, Changzhou, Wuxi, Suzhou (Jiangsu), Yancheng, Zhenjiang, Taizhou (Jiangsu), Suqian, Hangzhou, Ningbo, Jiaxing, Shaoxing, Wenzhou, Huzhou, Jinhua, Quzhou, Taizhou (Zhejiang), and Lishui. Block II is composed of 5 cities, namely Nantong, Lianyungang, Huai’an, Yangzhou, and Zhoushan. Block III contains 10 cities, namely Xuzhou, Hefei, Wuhu, Ma’anshan, Anqing, Bengbu, Chuzhou, Huangshan, Suzhou (Anhui), and Chizhou. And Block IV includes 7 cities, namely Huainan, Huaibei, Tongling, Fuyang, Lu’an, Bozhou, and Xuancheng.

According to the typology of Wasserman & Faust [57], in 2020, all cities in the YRD occupied primary positions, attributed to the high correlation of the spatial association network of digital economy innovation. With the advancement of the integration of the YRD, the correlation among cities has become increasingly close, and elements such as data, technology, talents, and funds are no longer limited to the local scope but flow among cities. The industries in diverse cities have established a cooperative innovation model propelled by the digital economy.

To further identify the relative characteristics of each block, the relative roles of each block are identified by this paper through the quadrant method. The results show that Block I is the primary position, represented by cities such as Shanghai, Hangzhou, and Nanjing. They resemble “star cities” within the realm of digital economy innovation. Benefiting from the solid internet industry foundation, and proactively bringing in global digital technologies, capital, and talents, a large number of internet enterprises, universities, and research institutions within the region collaborate intensively, yielding widespread radiating effects. Block II is the sycophant position, represented by cities such as Nantong, Lianyungang, and Zhoushan. These cities cooperate less with cities within the block, and their own digital economy innovation elements are more deprived by developed cities outside the block, while seldom receiving the industrial radiation of developed cities. Block III is the broker position, represented by cities such as Hefei and Wuhu. They maintain intimate links with cities beyond the block. However, an internally highly cohesive industrial collaborative network has not been established within the block yet. They play the role of resource integrators in the regional economy, build bridges between different blocks, and are hubs for the circulation of information and resources. Block IV is the isolated position, represented by cities such as Huainan, Huaibei, and Lu’an. These cities may be due to reasons such as remote geographical locations and weak industrial foundations, and have a relatively low participation in cross-regional economic cooperation, and are on the edge of the DEI network.

In light of the distribution of correlation relationships among diverse sectors, the inter-block density matrix can be computed as well to mirror the distribution of spillover effects across blocks (see Table 6).

Through calculation, the network density of the spatial correlation of DEI for cities in the YRD in 2020 was 0.792. Among the four blocks, if the network density of a particular block is higher than 0.792, it implies that the network density of this block exceeds the overall network density, and digital economy innovation is more concentrated in this block. Consequently, a value of 1 is assigned to the situation where the block network density is greater than the overall network density, and a value of 0 is assigned otherwise, thereby obtaining an image matrix. The image matrix can display the spillover effects among blocks more distinctly. The findings indicate that the spillover effects of Block I are predominantly reflected within Block I and Block III; the spillover effects of Block II are predominantly reflected in Block I; the spillover effects of Block III are predominantly reflected within Block III, Block I, and Block IV; the spillover effects of Block IV are predominantly reflected within Block IV and Block III.

### 4.2. Effects of the DEI Spatial Associative Network

#### 4.2.1. Effects of the Network Structure

A regression model for the effect analysis of the network structure was established. The descriptive statistics of each variable are shown in Table 7, and the model results are shown in Table 8.

In Table 8, Models (1)–(4) take the average of the DEI as the dependent variable, while Models (5)–(8) take the standard deviation of the DEI as the dependent variable. The Breusch-Pagan test results for all models show *p*-values greater than 0.1, failing to reject the null hypothesis of homoscedasticity at the 10% significance level. Nevertheless, to account for potential undetected heteroscedasticity, particularly given the limited sample size, we report heteroskedasticity-robust standard errors (HC3) for all estimations.

(1) The effect of the network structure on the DEI. In accordance with the regression outcomes of models (1)–(4) in Table 8, the regression coefficients for the network density, connectivity, hierarchy, and efficiency of the DEI spatial correlation are 0.569, 0.576, −0.576, and 0.572, respectively, all of which are significant at the 1% level. This estimation finding suggests that the network structure of the DEI spatial correlation in the YRD is significantly associated with the innovation level. Specifically, a 1% increase in network density, network connectedness, and network efficiency is associated with a 0.569%, 0.576%, and 0.572% rise in the digital economy innovation level of the YRD, respectively. In contrast, a 1% increase in network hierarchy corresponds to a 0.576% decrease in digital economy innovation performance.

(2) The effect of the network structure on the urban differences in the DEI. In light of the regression findings of models (5)–(8) in Table 8, the regression coefficients for the network density, connectivity, hierarchy, and efficiency of the DEI spatial correlation are −0.234, −0.224, 0.224, and −0.229, respectively, all being significant at the 1% significance level. This estimation finding suggests that the network structure of the DEI spatial correlation in the YRD is significantly associated with the urban disparities in the innovation level. Specifically, a 1% increase in network density, network connectedness, and network efficiency is associated with a reduction in inter-city disparity in digital economy innovation level by 0.234%, 0.224%, and 0.229%, respectively. In contrast, a 1% rise in network hierarchy corresponds to a 0.224% increase in such inter-city disparities.

#### 4.2.2. Effects of the Individual Network Features

Utilizing the panel data of 41 cities spanning from 2010 to 2020, a regression model for analyzing the effects of the individual network features was formulated. The descriptive statistics of each variable within the model are presented in Table 9.

In 2010–2020, the mean of the DEI for cities in the YRD is 0.691, the standard deviation is 0.142, the minimum is 0.112, and the maximum is 0.998. In 2020, the five cities boasting the highest digital economy innovation levels are Hangzhou, Shanghai, Ningbo, Jinhua, and Hefei, with their respective indexes being 0.998, 0.997, 0.983, 0.980, and 0.978. Leveraging their advantageous geographical positions, advanced scientific and technological levels, abundant talent resources, and complete industrial ecosystems, these cities have manifested robust competitiveness in the realm of digital economy innovation.

The DEI of cities like Huai’an, Huaibei, and Lianyungang is relatively low. Their indices are 0.712, 0.673, and 0.662, respectively. This can be ascribed to their comparatively traditional industrial structures, inadequate innovation investment, and feeble talent attraction.

This study employs two regression approaches—a baseline fixed-effects model and a dynamic panel model—to examine the impact of network centrality on digital economy innovation. The baseline fixed-effects model incorporates both city and time fixed effects, providing fundamental evidence on the relationships between variables. The dynamic panel model addresses path dependency in innovation and mitigates part of the endogeneity concerns by including the lagged dependent variable. To ensure the validity of statistical inference, we employ cluster-robust standard errors to account for potential heteroskedasticity. Furthermore, to avoid multicollinearity among network centrality measures, a grouped regression strategy is adopted, whereby highly correlated metrics—degree centrality, betweenness centrality, and closeness centrality—are introduced into separate models. This approach enhances the reliability of parameter estimates while maintaining model specification integrity.

The results of the panel regression models are presented in Table 10. Models (1) to (3) report the baseline regression results, while Models (4) to (6) present the dynamic panel regression estimates. The findings from the dynamic panel regressions remain substantively consistent with those of the baseline models.

The Hausman test results (*p* < 0.05 for models (1)–(3)) reject the null hypothesis, indicating that the fixed-effects model is preferred over the random-effects model for these specifications. The positive and statistically significant (at the 1% level) coefficients for all three centrality indicators indicate a strong positive association between a city’s centrality in the DEI spatial network and its level of digital economy innovation. Specifically, a 1% increase in IDG, ODG, BC, ICP, and OCP is associated with a respective increase in a city’s digital economy innovation level by 0.176%, 0.460%, 0.002%, 15.796%, and 30.080%.

### 4.3. Robustness Analysis

To verify the reliability of our findings, this section conducts robustness checks from the following two aspects: (1) tests concerning the selection of the binarization threshold; and (2) sensitivity analysis regarding the weighting of geographical versus institutional distance.

#### 4.3.1. Robustness Tests Based on Alternative Binarization Thresholds

The choice of the binarization threshold is critical when constructing the spatial adjacency matrix using the modified gravity model. Consistent with established practices in the literature [7,8,24], the baseline analysis uses the mean value of all elements in the gravity matrix as the threshold for binarization. However, to examine whether the network structure and subsequent regression results are sensitive to this choice, this study employs three alternative threshold scenarios for robustness testing. Scenario 1 uses the first quartile of all matrix elements as the threshold; Scenario 2 uses the median; and Scenario 3 uses the third quartile. These thresholds represent “denser,” “moderately dense,” and “sparser” network configurations, respectively, allowing for a comprehensive assessment of the stability of the results.

The Baseline scenario employs a weight combination of (*w*_1_ = 0.5, *w*_2_ = 0.5) with the mean as the threshold. Scenarios 1 to 3 utilize the same weight combination but vary the threshold, which is set at the first quartile, median, and third quartile, respectively. Scenarios 4 and 5, in contrast, share the mean as a common threshold and adopt different weight configurations of (*w*_1_ = 0.7, *w*_2_ = 0.3) and (*w*_1_ = 0.3, *w*_2_ = 0.7), respectively. Keeping all other parameters unchanged, we reconstructed the spatial connectedness network of digital economy innovation in the YRD for each year using each of the three alternative thresholds, recalculating key network structural metrics including density, connectedness, hierarchy, and efficiency.

The results, presented in Table 11, demonstrate that the core evolutionary characteristics of the network structure—specifically, the consistent year-on-year increase in network density and decrease in network hierarchy—remain robust across different threshold specifications.

The network structure variables and nodal characteristic measures constructed using the new thresholds were reintroduced as core explanatory variables and re-estimated using the original regression models (i.e., the models presented in Table 8 and Table 10), with a focus on whether the coefficient signs and significance levels of these core variables underwent fundamental changes. The regression results corresponding to Scenario 1 are presented in Table 12 and Table 13. Due to space constraints, the robustness check results for Scenarios 2 and 3 are reported in Table A1, Table A2, Table A5 and Table A6.

The findings demonstrate that, despite variations in the threshold, the main conclusions regarding the relationship between network structural characteristics and digital economy innovation remain qualitatively unaltered. Similarly, the primary findings concerning the relationship between nodal characteristics and the level of digital economy innovation also show no substantive change overall. These results collectively attest to the robustness of our research findings.

#### 4.3.2. Robustness Analysis Under Different Distance Weighting Scenarios

For simplicity, this study initially assigned equal weights (*w*_1_ = 0.5, *w*_2_ = 0.5) to geographical distance and institutional distance. To examine the sensitivity of the model results to variations in these weights and enhance the rationality and transparency of the model, a sensitivity analysis is conducted. Two alternative weighting scenarios are designed to simulate the relative importance of different driving factors: Scenario 4 emphasizes geographical factors (*w*_1_ = 0.7, *w*_2_ = 0.3), while Scenario 5 emphasizes institutional factors (*w*_1_ = 0.3, *w*_2_ = 0.7).

For each new weighting scenario, the composite distance is recalculated, and the spatial adjacency matrix is reconstructed using the original binarization threshold (the mean value). Key overall network structural metrics are subsequently computed and compared against those from the baseline model (0.5/0.5). The results, presented in Table A7, show that the core characteristics of network evolution—specifically, the consistent annual increase in network density and decrease in network hierarchy—also remain qualitatively unchanged.

Furthermore, the network structure variables and nodal characteristic variables calculated under the new weights are substituted into the regression models to observe changes in the coefficients and significance levels of the core explanatory variables. The core conclusions regarding network characteristics are robust, as shown in Table A3, Table A4, Table A5 and Table A6. Although specific connection strengths and centrality rankings are sensitive to model specifications, our main findings remain valid. This indicates that the core findings of this study are robust to variations in weight configurations. It also confirms empirically that the choice of equal weights, made initially for computational simplicity, does not compromise the reliability of the conclusions.

## 5. Discussion

### 5.1. Policy Drivers and Market Evolution Underlying Network Structural Changes

The evolutionary trends of the network structure revealed in this study are underpinned by profound policy drivers and market evolution dynamics. The sustained growth in network density and connectedness—ultimately achieving full connectivity—stems largely from the national strategy for promoting regional integration in the YRD. Since 2010, coordinated government efforts in digital infrastructure, including comprehensive high-speed broadband coverage, industrial internet identification systems, and collaborative data center planning, have substantially reduced physical barriers to inter-city digital factor flows [60]. Simultaneously, institutional innovations such as digital technology trading platforms and coordinated intellectual property protection have effectively lowered transaction costs for innovation cooperation [61,62], enabling previously isolated cities to rapidly integrate into the regional innovation network.

The marked decline in network hierarchy signals a fundamental transformation in regional innovation governance. This shift is driven by two key developments: Shanghai’s evolution from a primary “factor absorber” to a “radiative driver,” which actively fosters knowledge spillovers through mechanisms like the G60 Science and Technology Innovation Corridor and shared R&D platforms [63]; and the emergence of robust secondary centers (e.g., Nanjing, Hangzhou, and Hefei), forming a multipolar leadership pattern. This decentralization aligns with the resilience characteristics of complex adaptive systems [64,65]—reduced dependence on a single core node enhances the network’s capacity to withstand local shocks, thereby strengthening the stability of the overall regional innovation system.

The steady improvement in network efficiency demonstrates the synergy between market mechanisms and network evolution. As digital markets mature, innovation actors spontaneously optimize connections and eliminate redundant links to maximize collaborative benefits [66,67,68]. This “preferential attachment” mechanism channels resources toward pathways with higher innovation returns, thereby improving overall network allocation efficiency. Crucially, the parallel improvements in efficiency, density, and connectedness create a virtuous cycle: dense networks facilitate efficient connections, while efficient connections further reinforce network density. This co-evolutionary dynamic sustains the vitality of the YRD’s digital economy innovation system.

### 5.2. Theoretical Implications and Empirical Contributions

Our findings provide compelling empirical evidence for understanding digital economy innovation networks in the YRD while engaging substantially with established theories. The observed multi-center, non-hierarchical network structure—coupled with rising density/efficiency and declining hierarchy—strongly supports the core principles of complex adaptive system theory [64,65]. Rather than being dominated by a single core, the region has developed a resilient, co-evolving innovation ecosystem through “provincial engines” (Nanjing, Hefei, Hangzhou) that integrate internal resources, “regional bridges” (Wuhu, Chuzhou) that connect across sectors, and “knowledge hubs” (Shanghai, Ningbo) that attract advanced resources. This polycentric configuration not only enhances adaptive capacity [69,70] but also reveals a new regional innovation paradigm—shifting from “gradient transfer” to “multi-node symbiosis and networked collaboration.”

The strong positive correlation between centrality metrics and innovation performance—particularly the substantial gains by “bridge” cities (e.g., Wuhu, Chuzhou) through betweenness centrality—powerfully demonstrates the explanatory power of structural hole theory for regional innovation disparities [71,72]. By occupying strategic intermediary positions that control “gateways” for innovation flows, these cities integrate heterogeneous information, spark combinatorial innovations, and convert structural advantages into tangible performance. This mechanically clarifies that regional learning efficiency depends not merely on geographical proximity for knowledge spillovers, but more critically on topological positioning and bridge-building capacity within the network.

The broad positive effects of network density and connectedness on innovation levels—alongside their convergence effect on inter-city disparities—align perfectly with the fundamentals of innovation diffusion theory [73,74]: tightly interconnected networks drastically reduce friction in knowledge and technology transfer, enabling faster and more balanced regional diffusion. Conversely, network hierarchy’s suppression of overall innovation and amplification of urban gaps warns that excessive hierarchical structures hinder horizontal knowledge flows and mutual learning, which contradicts innovation’s inherent requirements for openness and flat interaction.

### 5.3. Limitations and Future Research Directions

This study identifies statistical associations rather than causal relationships between network features and digital economy innovation. Endogeneity concerns primarily arise from reverse causality (innovation potentially reshaping networks), omitted variables (e.g., policy environments simultaneously affecting networks and innovation), and measurement error. While robust standard errors improved inference reliability, causal identification strategies such as instrumental variables were constrained by sample size, necessitating cautious interpretation of results as correlational. Nevertheless, these findings offer valuable empirical evidence and theoretical insights into the network drivers of digital innovation, laying an important foundation for future research.

Despite employing fixed-effects and dynamic panel models that yielded consistent results, limitations persist. Data constraints prevent full resolution of endogeneity; although dynamic panels address some omitted variable and serial correlation concerns, reverse causality remains incompletely tackled. Future studies could identify more exogenous instruments (e.g., historical network structures or policy shocks) to strengthen causal claims. The exclusive focus on the YRD also warrants testing generalizability through expansion to other metropolitan regions.

Furthermore, while this study utilized city-level macrodata, incorporating firm-level microdata could illuminate how network effects operate through organizational decisions and knowledge absorption capacities, revealing macro-micro interactions between network structures and agent behaviors. The mechanisms linking network centrality to innovation also require empirical verification through mediators such as knowledge spillovers and resource flows. Finally, spatial econometric models could complement our analysis by examining the spatial spillover effects of network phenomena. In summary, while providing evidence of network effects on digital innovation, future research should address causality, expand geographical and analytical scales, investigate mechanisms, and incorporate spatial dependencies.

## 6. Conclusions and Policy Implications

### 6.1. Conclusions

Using panel data of 41 cities in the YRD, this paper examines digital economy innovation’s spatial correlation from a complex networks perspective. It constructs an adjusted gravity model to determine spatial correlations of DEI between cities and builds a network. An empirical study is also conducted on the structure and effects of the digital economy innovation spatial associative network. The main research findings are as follows:

First, in recent years, the digital economy innovation connections among cities in the YRD have become increasingly close, and the network has become more robust and less vulnerable. From 2010 to 2020, the network density of the DEI spatial correlation rose from 0.098 to 0.792, the connectedness rose from 0.359 to 1, the hierarchy dropped from 0.641 to 0, and the efficiency rose from 0.196 to 0.896.

Second, in terms of degree centrality, cities such as Nanjing and Hangzhou are the centers for gathering digital economy innovation resources in the region, while cities such as Tongling and Huai’an lack key factors like advanced industrial bases or preferential policies to attract external innovation resources. Wuhu plays a core role in the output of innovation achievements, and cities such as Fuyang and Bozhou have limited driving effects on the innovation development of other cities. In terms of betweenness centrality, cities such as Wuhu and Chuzhou are important nodes for the circulation of digital economy innovation information and resources, and have a strong control ability over the resource flow path. Cities such as Lianyungang, Zhoushan, and Huaibei are in relatively marginal or isolated positions and have no control over the resource flow. In terms of closeness centrality, cities such as Wuhu and Chuzhou are in the central position of the “supply circle” of information resources, and cities such as Huaibei and Lianyungang have weak abilities to obtain innovation information and integrate resources. Cities such as Wuhu and Chuzhou are more timely and convenient in information diffusion and resource output, and cities such as Zhoushan and Fuyang are relatively backward in terms of active information diffusion and resource output dominance.

Third, the 41 cities in the YRD are divided into four blocks. Block I (cities such as Shanghai and Nanjing) is the primary position and is the “star city” of digital economy innovation. Block II (cities such as Nantong and Lianyungang) is the sycophant position and receives fewer external innovation elements. Block III (cities such as Hefei and Wuhu) is the broker position and is a resource integrator. Block IV (cities such as Huainan and Huaibei) is in an isolated position and has a low degree of participation in cross-regional innovation cooperation.

Fourth, greater network density, connectedness, and efficiency are linked to a higher regional level of DEI and reduced urban disparities in DEI. Additionally, a city’s innovation level is positively correlated with its centrality in the network.

### 6.2. Policy Implications

This study embeds institutional distance into the gravity model to construct a spatial connectedness network, thereby characterizing the spatial pattern of digital economy innovation in the YRD from a complex network perspective and deepening the understanding of the relationship between “relational capital” and innovation performance. Future policy formulation should move beyond the conventional approach of “fostering advantaged entities” and place greater emphasis on cultivating and optimizing the overall structure of the regional innovation network. Specifically, policies should aim to strengthen the connective function of “bridge cities” and facilitate the network integration of non-core cities, thereby stimulating the endogenous innovative vitality of the entire region as a complex adaptive system.

First, core hubs and primary beneficiary blocks, such as Shanghai, Nanjing, and Hangzhou, should strengthen institutional coordination and promote high-quality knowledge spillovers. Policy measures should encourage these regional knowledge centers and provincial economic engines to evolve beyond simply absorbing resources and instead position themselves as organizers of regional innovation. Specifically, they should take the lead in establishing cross-regional innovation consortia and shared technology research and development platforms. Through creating institutionalized collaborative funding mechanisms, their knowledge dissemination can evolve from sporadic occurrences to systematic and sustained processes. Furthermore, promoting the co-development of innovation enclaves and pilot testing bases with neighboring cities will facilitate the circulation and sharing of talent, technology, and capital, thereby shifting the regional dynamic from polarization effects toward broader diffusion effects.

Second, bridge cities and broker blocks, including Wuhu, Chuzhou, and Hefei, should focus on enhancing connectivity while developing innovation initiation capabilities. Policies should support these cities that occupy strategic structural positions in transforming their informational advantages into genuine innovation capacity. Key initiatives include supporting the development and modernization of regional digital infrastructure, such as data exchange centers, intellectual property trading platforms, and technology transfer institutions, thereby formally enabling them to allocate resources across provincial boundaries. Additionally, establishing cross-provincial digital economy cooperation zones in areas adjacent to core cities, where unified market access and mutual standards recognition can be piloted, will help eliminate administrative barriers. This approach will elevate their role from passive resource conduits to active innovation integrators.

Third, peripheral cities and less integrated blocks, such as Nantong, Huainan, and Liuan, need targeted integration strategies to overcome marginalization challenges. For cities at risk of network marginalization, a dual approach is essential. The immediate priority should be coordinated regional efforts to address gaps in digital infrastructure, including high-speed broadband and computing centers, ensuring these cities possess the fundamental requirements for network integration. More importantly, a strength-based integration strategy should be implemented, encouraging these cities to avoid homogeneous competition and instead leverage their unique industrial foundations, such as specialized manufacturing or agriculture, to engage in complementary cooperation with core cities through scenario-driven digital economies. Through targeted talent programs and digital transformation subsidies, their capacity to absorb, adapt, and reinvent using external innovation resources can be significantly enhanced, gradually transforming them from network peripheries into specialized nodes.

## Figures and Tables

**Table 1:** The metropolitan areas in the Yangtze River Delta, China.

Metropolitan Area Name	Cities Included (Prefecture-Level and Above)
Shanghai metropolitan area	Shanghai; Changzhou, Nantong, Suzhou, Wuxi; Huzhou, Jiaxing, Ningbo, Zhoushan
Nanjing metropolitan area	Nanjing, Huai’an, Zhenjiang, Yangzhou, Chuzhou, Ma’anshan, Wuhu, Xuancheng
Hefei metropolitan area	Hefei, Lu’an, Wuhu, Huainan, Ma’anshan, Chuzhou, Bengbu
Hangzhou metropolitan area	Hangzhou, Shaoxing, Quzhou, Huzhou, Jiaxing, Huangshan
Ningbo metropolitan area	Ningbo, Taizhou, Zhoushan
Su-Xi-Chang metropolitan area	Suzhou, Wuxi, Changzhou

**Table 2:** Typology of positions in social network analysis.

	$\frac{\sum_{j\notin B_{k}}\sum_{i\in B_{k}}x_{jir}}{\sum_{j=1}^{g}\sum_{i=1}^{g}x_{jir}}\sim 0$	$\frac{\sum_{j\notin B_{k}}\sum_{i\in B_{k}}x_{jir}}{\sum_{j=1}^{g}\sum_{i=1}^{g}x_{jir}}>0$
$\frac{\sum_{i\in B_{k}}\sum_{j\in B_{k}}x_{ijr}}{\sum_{i\in B_{k}}\sum_{j=1}^{g}x_{ijr}}\geq \frac{g_{k}-1}{g-1}$	isolate	primary
$\frac{\sum_{i\in B_{k}}\sum_{j\in B_{k}}x_{ijr}}{\sum_{i\in B_{k}}\sum_{j=1}^{g}x_{ijr}}\leq \frac{g_{k}-1}{g-1}$	sycophant	broker

Source: Wasserman & Faust (1994) [57].

**Table 3:** Network centrality metrics of the digital economy innovation spatial correlation in the Yangtze River Delta for the year 2020.

City	Degree Centrality		Betweenness Centrality	Closeness Centrality	
	In-Degree	Out-Degree		In-Closeness	Out-Closeness
Shanghai	0.925	0.700	2.842	0.023	0.019
Nanjing	1.000	0.925	20.750	0.025	0.023
Wuxi	0.925	0.850	9.009	0.023	0.022
Xuzhou	0.900	0.875	15.746	0.023	0.022
Changzhou	0.900	0.850	8.226	0.023	0.022
Suzhou, Jiangsu	0.925	0.825	8.065	0.023	0.021
Nantong	0.825	0.750	5.142	0.021	0.020
Lianyungang	0.300	0.600	0.000	0.015	0.018
Huai’an	0.400	0.775	1.055	0.016	0.020
Yancheng	0.900	0.800	6.897	0.023	0.021
Yangzhou	0.625	0.825	3.161	0.018	0.021
Zhenjiang	0.875	0.875	8.385	0.022	0.022
Taizhou, Jiangsu	0.900	0.825	7.282	0.023	0.021
Suqian	0.975	0.825	11.974	0.024	0.021
Hangzhou	1.000	0.825	9.906	0.025	0.021
Ningbo	0.900	0.625	1.531	0.023	0.018
Wenzhou	0.875	0.700	2.010	0.022	0.019
Jiaxing	0.925	0.775	3.896	0.023	0.020
Huzhou	0.925	0.825	6.018	0.023	0.021
Shaoxing	0.925	0.775	3.896	0.023	0.020
Jinhua	1.000	0.825	9.906	0.025	0.021
Quzhou	0.900	0.825	5.441	0.023	0.021
Zhoushan	0.500	0.525	0.000	0.017	0.017
Taizhou, Zhejiang	0.775	0.600	0.693	0.020	0.018
Lishui	0.900	0.775	3.473	0.023	0.020
Hefei	1.000	0.925	22.613	0.025	0.023
Wuhu	1.000	0.975	26.314	0.025	0.024
Bengbu	0.925	0.900	16.592	0.023	0.023
Huainan	0.575	0.850	5.639	0.018	0.022
Ma’anshan	0.975	0.950	22.272	0.024	0.024
Huaibei	0.375	0.500	0.000	0.015	0.017
Tongling	0.400	0.850	2.852	0.016	0.022
Anqing	0.925	0.900	16.564	0.023	0.023
Huangshan	0.800	0.900	11.851	0.021	0.023
Chuzhou	1.000	0.950	24.649	0.025	0.024
Fuyang	0.400	0.500	0.192	0.016	0.017
Suzhou, Anhui	0.825	0.850	11.666	0.021	0.022
Lu’an	0.425	0.700	1.745	0.016	0.019
Bozhou	0.400	0.500	0.192	0.016	0.017
Chizhou	0.825	0.900	12.567	0.021	0.023
Xuancheng	0.625	0.950	9.987	0.018	0.024

**Table 4:** Centrality Metrics Correlation analysis between per capita GDP and network centrality metrics.

Metrics	Pooled_Pearson	Between_Pearson	Within_Pearson	Pooled_Spearman	Between_Spearman	Within_Spearman
in-degree	0.604 ***	0.594 ***	0.779 ***	0.614 ***	0.590 ***	0.817 ***
out-degree	0.510 ***	0.526 ***	0.790 ***	0.531 ***	0.555 ***	0.836 ***
betweenness centrality	0.188 ***	0.381 **	−0.231 ***	0.488 ***	0.527 ***	−0.022
in-closeness	0.587 ***	0.612 ***	0.750 ***	0.598 ***	0.638 ***	0.809 ***
out-closeness	0.484 ***	0.496 ***	0.799 ***	0.482 ***	0.523 ***	0.856 ***

Note: *** *p* < 0.01, ** *p* < 0.05.

**Table 5:** Block model analysis of the digital economy innovation spatial correlation network for the year 2020.

Block	Number of Receiving Relationships				Number of Members	Number of Relationships Received from Outside	Number of Relationships Spilling out of the Block	Expected Proportion of Internal Relationships (%)	Actual Proportion of Internal Relationships (%)	Block Receiving Ratio
	I	II	III	IV						
Block I	342	69	173	17	19	356	259	0.450	0.569	0.274
Block II	89	14	34	2	5	92	125	0.100	0.101	0.071
Block III	188	20	90	67	10	277	275	0.225	0.247	0.213
Block IV	79	3	70	42	7	86	152	0.150	0.216	0.066

**Table 6:** Inter-block spillover effects: density matrix and image matrix.

Block	Density Matrix				Image Matrix			
	Block I	Block II	Block III	Block IV	Block I	Block II	Block III	Block IV
Block I	1.000	0.726	0.911	0.128	1	0	1	0
Block II	0.937	0.700	0.680	0.057	1	0	0	0
Block III	0.989	0.400	1.000	0.957	1	0	1	1
Block IV	0.594	0.086	1.000	1.000	0	0	1	1

Note: In the image matrix, 1 indicates the existence of an association relationship where the row points to the column, while 0 indicates the absence of such an association relationship.

**Table 7:** Descriptive statistics of variables for network structure effect analysis.

Variable	Variable Description	Mean	Std.Dev	Min.	Max.
DAV	Digital Economy Innovation Average Value	0.696	0.142	0.470	0.874
DST	Digital Economy Innovation Standard Deviation	0.172	0.058	0.090	0.254
ND	Network Density	0.411	0.245	0.098	0.792
NC	Network Connectivity	0.803	0.239	0.359	1.000
NH	Network Hierarchy	0.197	0.239	0.000	0.641
NE	Network Efficiency	0.593	0.248	0.196	0.896

**Table 8:** Regression results on the effect of overall network structure on digital economy innovation.

Model	(1)	(2)	(3)	(4)	(5)	(6)	(7)	(8)
*CONS*	0.462 *** (0.025)	0.234 *** (0.045)	0.810 *** (0.017)	0.357 *** (0.005)	0.268 *** (0.007)	0.352 *** (0.028)	0.128 *** (0.010)	0.308 *** (0.010)
*ND*	0.569 *** (0.054)				−0.234 *** (0.012)			
*NC*		0.576 *** (0.056)				−0.224 *** (0.034)		
*NH*			−0.576 *** (0.056)				0.224 *** (0.034)	
*NE*				0.572 *** (0.007)				−0.229 *** (0.016)
Breusch-Pagan Test (*p* value)	0.428	0.377	0.377	0.599	0.082	0.221	0.221	0.396
R^2^	0.959	0.937	0.937	0.998	0.981	0.863	0.863	0.970

Note: The dependent variable of Models (1)–(4) is Digital Economy Innovation Average Value (DAV), while the dependent variable of Models (5)–(8) is Digital Economy Innovation Standard Deviation (DST); *** *p* < 0.01; Heteroskedasticity-robust standard errors (HC3) are reported in parentheses.

**Table 9:** Descriptive statistics of variables for node-level effect analysis.

Variable	Variable Description	Mean	Std.	Min.	Max.
*DL*	Digital Economy Level	0.696	0.226	0.112	0.998
*IDG*	In-Degree Centrality	0.411	0.325	0.000	1.000
*ODG*	Out-Degree Centrality	0.411	0.265	0.000	0.975
*BC*	Betweenness Centrality	19.253	37.403	0.000	254.386
*ICP*	In-Closeness Centrality	0.014	0.008	0.000	0.025
*OCP*	Out-Closeness Centrality	0.013	0.006	0.000	0.024

**Table 10:** Panel regression results of node centrality on digital economy innovation level.

Model	(1)	(2)	(3)	(4)	(5)	(6)
*DL*(−1)				0.312 *** (0.057)	0.270 *** (0.066)	0.131 *** (0.038)
*IDG*	0.176 *** (0.051)			0.251 *** (0.042)		
*ODG*	0.460 *** (0.097)			0.355 *** (0.074)		
*BC*		0.002 *** (0.000)			0.002 *** (0.000)	
*ICP*			15.796 *** (1.661)			13.874 *** (1.439)
*OCP*			30.080 *** (2.848)			30.147 *** (2.693)
City Fixed Effect	YES	YES	YES	YES	YES	YES
Year Fixed Effect	YES	YES	YES	YES	YES	YES
VIF	4.032		3.974			
Breusch-Pagan Test (*p* value)	0.000	0.0169	0.000			
Hausman (*p* value)	0.000	0.015	0.000			
Adjusted R^2^	0.262	0.193	0.596	0.462	0.287	0.684

Note: *** *p* < 0.01. The dependent variable is the Digital Economy Level (DL). DL(−1) denotes the one-period lagged value of the Digital Economy Level. Values in parentheses are cluster-robust standard errors, with clustering at the city level.

**Table 11:** Robustness check: network structural metrics under alternative binarization thresholds.

Year	The Baseline Scenario				Scenario 1				Scenario 2				Scenario 3			
	ND	NC	NH	NE	ND	NC	NH	NE	ND	NC	NH	NE	ND	NC	NH	NE
2010	0.098	0.359	0.641	0.196	0.320	0.713	0.287	0.510	0.137	0.443	0.557	0.266	0.062	0.176	0.824	0.105
2011	0.131	0.468	0.532	0.272	0.428	0.805	0.195	0.613	0.179	0.543	0.457	0.346	0.078	0.286	0.714	0.158
2012	0.160	0.560	0.440	0.333	0.457	0.829	0.171	0.640	0.200	0.601	0.399	0.386	0.090	0.410	0.590	0.199
2013	0.215	0.737	0.263	0.449	0.626	0.976	0.024	0.800	0.290	0.780	0.220	0.522	0.126	0.587	0.413	0.302
2014	0.301	0.805	0.195	0.534	0.779	1.000	0.000	0.890	0.407	0.854	0.146	0.624	0.180	0.732	0.268	0.403
2015	0.415	0.951	0.049	0.672	0.902	1.000	0.000	0.951	0.536	0.951	0.049	0.743	0.249	0.829	0.171	0.499
2016	0.525	0.976	0.024	0.747	0.967	1.000	0.000	0.984	0.682	0.976	0.024	0.828	0.323	0.951	0.049	0.605
2017	0.546	0.976	0.024	0.758	0.977	1.000	0.000	0.988	0.675	0.976	0.024	0.825	0.332	0.927	0.073	0.602
2018	0.681	1.000	0.000	0.840	1.000	1.000	0.000	1.000	0.838	1.000	0.000	0.919	0.443	0.976	0.024	0.696
2019	0.654	1.000	0.000	0.826	1.000	1.000	0.000	1.000	0.799	1.000	0.000	0.900	0.426	1.000	0.000	0.695
2020	0.792	1.000	0.000	0.896	1.000	1.000	0.000	1.000	0.895	1.000	0.000	0.948	0.510	1.000	0.000	0.749

**Table 12:** Robustness of network structure effects: regression results under low threshold (Scenario 1).

Model	(1)	(2)	(3)	(4)	(5)	(6)	(7)	(8)
*CONS*	0.289 *** (0.019)	−0.413 (0.213)	0.769 *** (0.030)	0.057 (0.061)	0.332 *** (0.015)	0.597 *** (0.107)	0.144 *** (0.014)	0.422 *** (0.037)
*ND*	0.530 *** (0.031)				−0.208 *** (0.023)			
*NC*		1.182 *** (0.230)				−0.453 *** (0.115)		
*NH*			−1.182 *** (0.230)				0.453 *** (0.115)	
*NE*				0.750 *** (0.072)				−0.293 *** (0.045)
Breusch-Pagan Test (*p* value)	0.127	0.238	0.238	0.328	0.073	0.179	0.179	0.163
R^2^	0.966	0.748	0.748	0.931	0.904	0.668	0.668	0.861

Note: The dependent variable of Models (1)–(4) is Digital Economy Innovation Average Value (DAV), while the dependent variable of Models (5)–(8) is Digital Economy Innovation Standard Deviation (DST); *** *p* < 0.01; Heteroskedasticity-robust standard errors (HC3) are reported in parentheses. For results under other scenarios, see Table A1 and Table A2.

**Table 13:** Robustness of node-level effects: regression results under low threshold (Scenario 1).

Model	(1)	(2)	(3)	(4)	(5)	(6)
*DL*(−1)				0.037 (0.046)	0.308 *** (0.072)	0.070 * (0.041)
*IDG*	0.307 *** (0.024)			0.296 *** (0.025)		
*ODG*	0.318 *** (0.037)			0.282 *** (0.043)		
*BC*		0.002 *** (0.001)			0.003 *** (0.001)	
*ICP*			19.221 *** (1.555)			18.423 *** (1.717)
*OCP*			2.603 (5.658)			3.285 (5.916)
City Fixed Effects	YES	YES	YES	YES	YES	YES
Year Fixed Effects	YES	YES	YES	YES	YES	YES
VIF	3.550		3.250			
Breusch-Pagan Test (*p* value)	0.000	0.000	0.079			
Hausman (*p* value)	0.000	0.015	0.000			
Adjusted R^2^	0.596	0.090	0.550	0.548	0.20	0.526

Note: *** *p* < 0.01, * *p* < 0.1. The dependent variable is the Digital Economy Level (DL). DL(−1) denotes the one-period lagged value of the Digital Economy Level. Values in parentheses are cluster-robust standard errors, with clustering at the city level. For results under other scenarios, see Table A5 and Table A6.

## Data Availability

The raw data supporting the conclusions of this article will be made available by the authors on request.

## Abbreviations

The following abbreviations are used in this manuscript:

YRD	The Yangtze River Delta, China
AI	Artificial intelligence
VAR	Vector Autoregression
DEI	The digital economy innovation index
IRIEDEC	The Index of Regional Innovation and Entrepreneurship in the Digital Economy in China
R_ij_	The strength of the digital economy innovation connection from city i to city j
K_ij_	The gravitational coefficient
D	The revised distance between cities
D_1_	The geographical distance between cities
D_2_	The institutional distance between cities
ND	Network density
NC	Network connectivity
NH	Network hierarchy
NE	Network efficiency
Cd	Degree centrality
Cb	Betweenness centrality
Cc	Closeness centrality
OLS	Ordinary Least Squares
DAV	Digital Economy Innovation Average Value
DST	Digital Economy Innovation Standard Deviation
DL	Digital Economy Level
DL(−1)	The one-period lagged value of the Digital Economy Level
IDG	In-Degree Centrality
ODG	Out-Degree Centrality
BC	Betweenness Centrality
ICP	In-Closeness Centrality
OCP	Out-Closeness Centrality

## Appendix A

**Table A1:** Robustness of network structure effects: regression results under middle threshold (Scenario 2).

Model	(1)	(2)	(3)	(4)	(5)	(6)	(7)	(8)
*CONS*	0.441 *** (0.021)	0.148 * (0.052)	0.809 *** (0.018)	0.317 *** (0.006)	0.276 *** (0.006)	0.386 *** (0.032)	0.128 *** (0.011)	0.324 *** (0.011)
*ND*	0.497 *** (0.030)				−0.203 *** (0.008)			
*NC*		0.662 *** (0.063)				−0.258 *** (0.039)		
*NH*			−0.662*** (0.063)				0.258 *** (0.039)	
*NE*				0.571 *** (0.011)				−0.228 *** (0.017)
R^2^	0.979	0.933	0.933	0.998	0.986	0.863	0.863	0.968

Note: The dependent variable of Models (1)–(4) is Digital Economy Innovation Average Value (DAV), while the dependent variable of Models (5)–(8) is Digital Economy Innovation Standard Deviation (DST); *** *p* < 0.01, * *p* < 0.1; Heteroskedasticity-robust standard errors (HC3) are reported in parentheses.

**Table A2:** Robustness of network structure effects: regression results under high threshold (Scenario 3).

Model	(1)	(2)	(3)	(4)	(5)	(6)	(7)	(8)
*CONS*	0.476 *** (0.028)	0.368 *** (0.025)	0.827 *** (0.013)	0.420 *** (0.008)	0.263 *** (0.008)	0.301 *** (0.018)	0.121 *** (0.010)	0.283 *** (0.006)
*ND*	0.858 *** (0.092)				−0.355 *** (0.022)			
*NC*		0.459 *** (0.033)				−0.180 *** (0.024)		
*NH*			−0.459 *** (0.033)				0.180 *** (0.024)	
*NE*				0.606 *** (0.014)				−0.244 *** (0.013)
R^2^	0.942	0.968	0.968	0.997	0.977	0.907	0.907	0.983

Note: The dependent variable of Models (1)–(4) is Digital Economy Innovation Average Value (DAV), while the dependent variable of Models (5)–(8) is Digital Economy Innovation Standard Deviation (DST); *** *p* < 0.01; Heteroskedasticity-robust standard errors (HC3) are reported in parentheses.

**Table A3:** Robustness of network structure effects: regression results under Scenario 4.

Model	(1)	(2)	(3)	(4)	(5)	(6)	(7)	(8)
*CONS*	0.472 *** (0.026)	0.295 *** (0.071)	0.808 *** (0.018)	0.380 *** (0.005)	0.264 *** (0.007)	0.328 *** (0.037)	0.128 *** (0.011)	0.299 *** (0.010)
*ND*	0.534 *** (0.051)				−0.219 *** (0.012)			
*NC*		0.513 *** (0.082)				−0.200 *** (0.044)		
*NH*			−0.513 *** (0.082)				0.200 *** (0.044)	
*NE*				0.536 *** (0.007)				−0.214 *** (0.016)
R^2^	0.956	0.926	0.926	0.998	0.979	0.853	0.853	0.971

Note: The dependent variable of Models (1)–(4) is Digital Economy Innovation Average Value (DAV), while the dependent variable of Models (5)–(8) is Digital Economy Innovation Standard Deviation (DST); *** *p* < 0.01; Heteroskedasticity-robust standard errors (HC3) are reported in parentheses.

**Table A4:** Robustness of network structure effects: regression results under Scenario 5.

Model	(1)	(2)	(3)	(4)	(5)	(6)	(7)	(8)
*CONS*	0.449 *** (0.025)	0.206 *** (0.043)	0.809 *** (0.017)	0.337 *** (0.008)	0.273 *** (0.007)	0.362 *** (0.027)	0.128 *** (0.011)	0.315 *** (0.011)
*ND*	0.624 *** (0.054)				−0.256 *** (0.012)			
*NC*		0.603 *** (0.054)				−0.234 *** (0.035)		
*NH*			−0.603 *** (0.054)				0.234 *** (0.035)	
*NE*				0.609 *** (0.012)				−0.243 *** (0.018)
R^2^	0.965	0.942	0.942	0.998	0.985	0.860	0.860	0.966

Note: The dependent variable of Models (1)–(4) is Digital Economy Innovation Average Value (DAV), while the dependent variable of Models (5)–(8) is Digital Economy Innovation Standard Deviation (DST); *** *p* < 0.01; Heteroskedasticity-robust standard errors (HC3) are reported in parentheses.

**Table A5:** Robustness of node-level effects: benchmark regression results under alternative binarization thresholds and distance weights (Scenarios 2–5).

Explanatory Variable	Scenario 2			Scenario 3			Scenario 4			Scenario 5		
	(1)	(2)	(3)	(1)	(2)	(3)	(1)	(2)	(3)	(1)	(2)	(3)
*IDG*	0.225 *** (0.034)			−0.196 * (0.102)			0.125 *** (0.047)			0.281 *** (0.060)		
*ODG*	0.544 *** (0.074)			0.363 ** (0.177)			0.623 *** (0.106)			0.275 ** (0.109)		
*BC*		0.002 *** (0.0004)			0.0001 (0.0001)			0.002 *** (0.0004)			0.002 *** (0.0004)	
*ICP*			15.021 *** (1.453)			2.802 (1.713)			13.592 *** (1.338)			18.374 *** (2.076)
*OCP*			27.421 *** (2.150)			19.854 *** (6.059)			38.298 *** (2.884)			25.667 *** (3.467)
City Fixed Effect	YES	YES	YES	YES	YES	YES	YES	YES	YES	YES	YES	YES
Year Fixed Effect	YES	YES	YES	YES	YES	YES	YES	YES	YES	YES	YES	YES
Observations	451	451	451	451	451	451	451	451	451	451	451	451
Adjusted R^2^	0.537	0.202	0.71	−0.101	−0.127	−0.055	0.336	0.127	0.647	0.22	0.214	0.579
VIF	3.74		3.76	5.1		4.04	3.70		3.88	4.86		4.43
Hausman (*p* value)	0.000	0.000	0.000	0.000	0.000	0.000	0.000	0.273	0.000	0.000	0.069	0.000

Note: Scenario 2 and 3 employ an identical weight combination (*w*_1_ = 0.5, *w*_2_ = 0.5), with their thresholds set at the median and third quartile, respectively. In contrast, Scenario 4 and 5 share a common threshold (the mean), utilizing weight configurations of (*w*_1_ = 0.7, *w*_2_ = 0.3) and (*w*_1_ = 0.3, *w*_2_ = 0.7), respectively. *** *p* < 0.01, ** *p* < 0.05, * *p* < 0.1. The dependent variable is Digital Economy Level(DL). Values in parentheses are cluster-robust standard errors, with clustering at the city level.

**Table A6:** Robustness of node-level effects: dynamic panel regression results under alternative binarization thresholds and distance weights (Scenarios 2–5).

Explanatory Variable	Scenario 2			Scenario 3			Scenario 4			**Scenario 5**		
	(1)	(2)	(3)	(1)	(2)	(3)	(1)	(2)	(3)	**(1** **)**	**(2)**	**(3)**
*DL*(−1)	0.187 *** (0.040)	0.262 *** (0.069)	0.090 *** (0.033)	0.374 *** (0.070)	0.383 *** (0.070)	0.345 *** (0.071)	0.278 *** (0.047)	0.268 *** (0.066)	0.124 *** (0.033)	0.334 *** (0.061)	0.273 *** (0.065)	0.136 *** (0.045)
*IDG*	0.253 *** (0.028)			−0.107 (0.075)			0.197 *** (0.038)			0.356 *** (0.052)		
*ODG*	0.433 *** (0.058)			0.152 (0.113)			0.494 *** (0.081)			0.163 * (0.083)		
*BC*		0.002 *** (0.0004)			0.00001 (0.0001)			0.002 *** (0.0004)			0.002 *** (0.0004)	
*ICP*			14.383 *** (1.440)			1.268 (1.529)			12.730 *** (1.193)			16.812 *** (1.970)
*OCP*			24.996 *** (2.139)			9.986 ** (4.011)			36.447 *** (2.416)			24.653 *** (3.423)
City Fixed Effect	YES	YES	YES	YES	YES	YES	YES	YES	YES	YES	YES	YES
Year Fixed Effect	YES	YES	YES	YES	YES	YES	YES	YES	YES	YES	YES	YES
Observations	410	410	410	410	410	410	410	410	410	410	410	410
Adjusted R^2^	0.638	0.271	0.731	0.039	0.036	0.051	0.499	0.262	0.720	0.430	0.300	0.630

Note: Scenario 2 and 3 employ an identical weight combination (*w*_1_ = 0.5, *w*_2_ = 0.5), with their thresholds set at the median and third quartile, respectively. In contrast, Scenario 4 and 5 share a common threshold (the mean), utilizing weight configurations of (*w*_1_ = 0.7, *w*_2_ = 0.3) and (*w*_1_ = 0.3, *w*_2_ = 0.7), respectively. *** *p* < 0.01, ** *p* < 0.05, * *p* < 0.1. The dependent variable is Digital Economy Level(DL). Values in parentheses are cluster-robust standard errors, with clustering at the city level.

**Table A7:** Robustness of network evolution trends: structural metrics under alternative distance weights (Scenarios 4–5).

Year	Scenario 4				Scenario 5			
	ND	NC	NH	NE	ND	NC	NH	NE
2010	0.094	0.244	0.756	0.160	0.107	0.397	0.603	0.223
2011	0.122	0.449	0.551	0.257	0.134	0.488	0.512	0.282
2012	0.154	0.518	0.482	0.311	0.162	0.601	0.399	0.35
2013	0.209	0.732	0.268	0.443	0.224	0.695	0.305	0.436
2014	0.301	0.78	0.22	0.523	0.302	0.829	0.171	0.544
2015	0.418	0.927	0.073	0.665	0.401	0.951	0.049	0.663
2016	0.546	0.976	0.024	0.758	0.499	0.976	0.024	0.733
2017	0.566	0.976	0.024	0.769	0.524	1.000	0.000	0.753
2018	0.709	1.000	0.000	0.855	0.646	1.000	0.000	0.822
2019	0.684	1.000	0.000	0.842	0.619	1.000	0.000	0.808
2020	0.824	1.000	0.000	0.912	0.738	1.000	0.000	0.869

Note: Scenario 4 and 5 share a common threshold (the mean), utilizing weight configurations of (*w*_1_ = 0.7, *w*_2_ = 0.3) and (*w*_1_ = 0.3, *w*_2_ = 0.7), respectively.
